# Supplementary material for: Demonstration of in vivo engineered tandem duplications of varying sizes using CRISPR and recombinases in Drosophila melanogaster
Source: G3 (Bethesda). 2023 Jul 18;13(10):jkad155. doi: 10.1093/g3journal/jkad155 (PMC10542505; doi:10.1093/g3journal/jkad155)
Supplement: jkad155_Supplementary_Data [file jkad155_supplementary_data.zip › Figure_S1_G3-2023-404347.pdf]

**A**

Probe: *w.* Digest: XhoI or XhoI + KpnI

| genotype | Expected XhoI | Expected XhoI+KpnI |
|---|---|---|
| y w hs-flp;Sco/CyO (uninserted) | 10.0 kb | 6.2 |
| MX2.3 | 10.0, 19.4 | 6.2, 15.6 |
| MX5.1 | 10.0, 27.1 | 6.2, 9.9, 13.5 |
| MX6.1 | 10.0, 7.7,10.5 | 6.2, 4.6, 6.8 |
| MX10.1 | 10.0, +8.7 | 6.2, +8.7 |

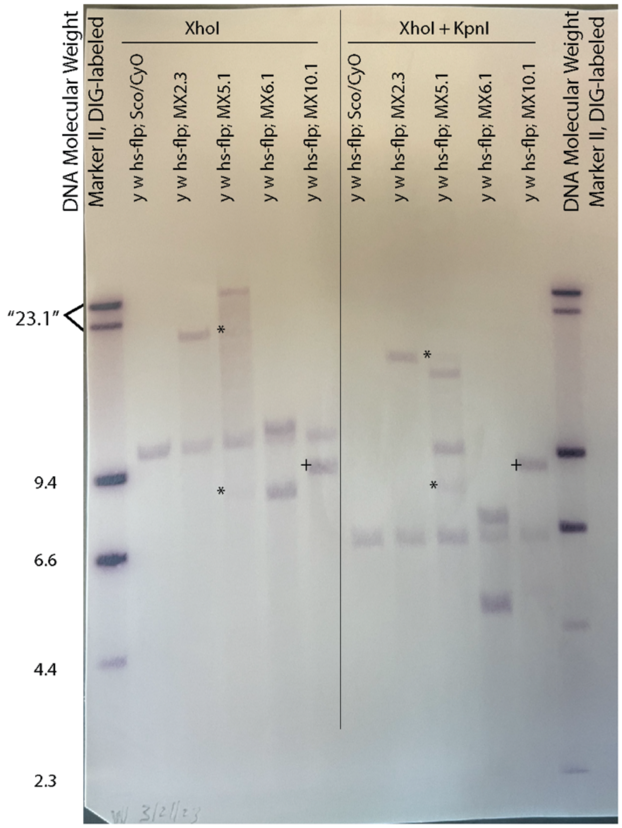


**B**

Probe: *Adh*. Digest: NheI + PmeI.

| genotype | Expected |
|---|---|
| 55821 (uninserted) | 19.1 kb |
| MX2.3 | 22.7 |
| MX5.1 | 30.4 (solo w: 22.7) |
| MX6.1 | 30.4 (solo w: 22.7) |
| MX10.1 | 19.1 |
| TD6/2 | 27.8 |
| TD10/2 | 19.1, 20.7 |
| TD6/5 | 23.4 |
| TD10/5 | 19.1, 16.3 |

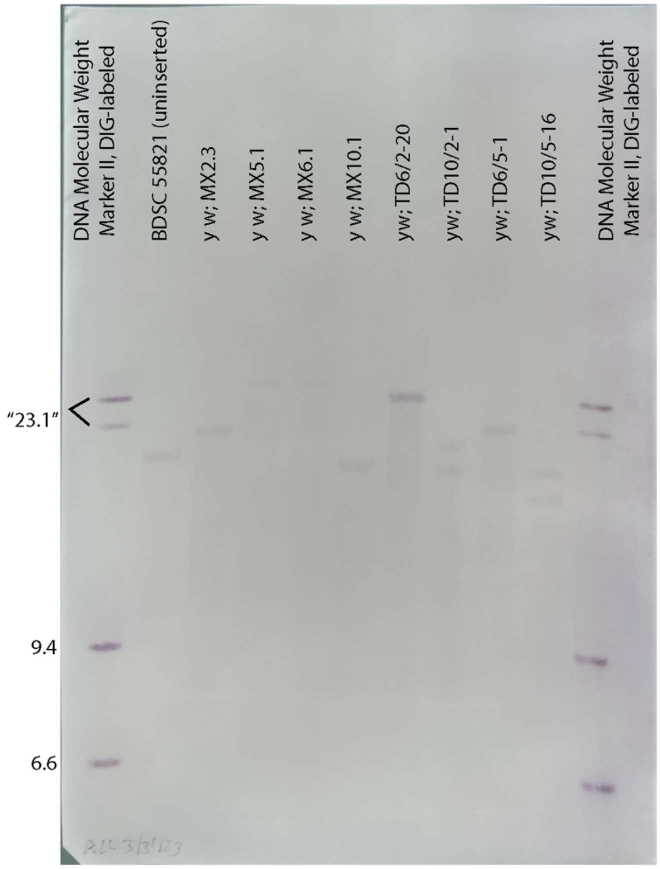

**Figure S1: Southern (genomic DNA) blot verification of *Adh* region structure.**

**A)** Size of *w*-containing fragments in the CRISPR insertion lines (including the endogenous *w* locus). The table shows the predicted band sizes for each genotype. Overall, the expected structures were observed. Three aspects of the blot data were unanticipated and are discussed below, but the analysis does not change the overall conclusion.

- The top ("23.1 kb") band of the digoxigenin-labeled marker separates into two bands over long gel run times, which is an undocumented aspect of the product (DNA Molecular Weight Marker II, DIG-Labelled (Roche/MilliporeSigma 11218590910)). We speculate that the digoxigenin labeling of the 23.1 kb fragment is variable, with an effect on the migration rate of the bands. If so, the lower of the two bands marked "23.1 kb" would be more accurate.
- The predicted size of one MX10 fragment (marked with +) did not match the 6.1kb size predicted from the reference genome. This led us to analyze other *Adh* haplotypes from the DSPR whole-genome assemblies (Chakraborty et al. 2019) and identify a closer match to the haplotype of strain BDSC 55821: this strain is most similar to the genome of A1 (Canton-S). The anomalous band is then readily explained by a polymorphic XhoI site in the *D. melanogaster* population. The XhoI site is absent in strains A1, A2, and ORE, predicting an 8.7kb fragment upon *FRT-w* insertion in strain MX10. A ~8.7kb fragment is observed. The A1-like haplotype is also consistent with the banding pattern seen in the *Adh* blot.
- Faint bands were observed (marked with *) in genotype *y w hs-Flp; MX5.1*. The MX5.1 insertion carries two w-FRT insertions, as determined by phenotypic and ddPCR data. All expected bands occur, with greater intensity. The faint bands match the sizes predicted if the two-*w*-copy insertion in this line recombined down to one copy in some cells. Somatic recombination of two *w* copies down to one is plausible given that this strain carries *hs-Flp*. Consistent with this, faint bands were not observed in a test blot (not shown) that used genomic DNA from strain *y w; MX5.1* that lacks *hs-Flp*. Somatic recombination could also have occurred in *y w hs-Flp; MX6.1*, which also carries two *w* copies based on the phenotypic and ddPCR data. However, this could not be detected as the digest pattern in this strain is predicted to be identical for one or two inserted *w* copies.

**B)** Size of *Adh*-containing fragments in insertion and tandem-duplication lines. Blot is probed with three adjacent DIG-labeled *Adh* fragments. Expected product sizes are given in the table, based on restriction sites in the DSPR A1 (Canton-S) assembly. A1 contains a NheI site not present in the reference genome. Overall, the pattern of bands matches the relative size predictions. Details of the analysis follow.

- PmeI was included in the digest mix in case the polymorphic NheI site was absent, as in the reference genome. The banding pattern is consistent with the A1 haplotype, in which NheI sites flank the *Adh* region.
- Estimating the absolute size is made somewhat uncertain because the reference band in the ladder, the "23.1 kb" band, has separated into two bands. If this were due to uneven DIG labeling of the product, we would expect the band with fewer digoxigenin side-groups to be more accurate, i.e., the lower band. This lower band migrates at

about the same size as MX2.3 and TD6/5, which were predicted to have sizes of 23 kb. Conversely, no predicted structure produced bands that are consistent with the upper "23.1 kb" band in the DIG-labeled standard being accurate.

- Regardless of the performance of the ladder, every fragment detected with the *Adh* probe is consistent with the predicted sizes. These data are also consistent with the structure and copy number determined using junction PCR and digital-PCR.
