# Supplementary material for: Demonstration of in vivo engineered tandem duplications of varying sizes using CRISPR and recombinases in Drosophila melanogaster
Source: G3 (Bethesda). 2023 Jul 18;13(10):jkad155. doi: 10.1093/g3journal/jkad155 (PMC10542505; doi:10.1093/g3journal/jkad155)
Supplement: jkad155_Supplementary_Data [file jkad155_supplementary_data.zip › Figure_S2_G3-2023-404347.pdf]

Figure S2A

```
NT_033779          TGGCCCTACAACCACTTATTATTCAGTTGACTGTTAAATTGCTTGAGTTATTTCCACAAC   60
BDSC 55821         ............................................................   60
BG55821 (fast)     ............................................................   60
HarMX2 construct   TAGGAACTTC..................................................   60
MX2.3 PCR product  TAGGAACTTC..................................................   60

NT_033779          TTGATGAAGTTTGTTATTGATAGAGCCAATTTTATAATACTAATCTAACACGAGATTCAG  120
BDSC 55821         ............................................................  120
BG55821 (fast)     ............................................................  120
HarMX2 construct   ............................................................  120
MX2.3 PCR product  ............................................................  120

NT_033779          TTAATGTTTCATTAAAATAAAAGTTTAGTTAGAAGAATATACTTCTAGATTTTAAAATTT  180
BDSC 55821         ............................................................  180
BG55821 (fast)     ............................................................  180
HarMX2 construct   ............................................................  180
MX2.3 PCR product  ............................................................  180

NT_033779          ATACTTTCTAAGAGCTTTTTGCAATTATATGGTTTGTGTTTCCGCTAAGTTATAAATTAG  240
BDSC 55821         ............................................................  240
BG55821 (fast)     ............................................................  240
HarMX2 construct   ............................................................  240
MX2.3 PCR product  ............................................................  240

NT_033779          CTGATCAGCTTTTTAGATTTCTCTAAAGAAAAGTTAGTTT------------------TA  282
BDSC 55821         ......................A....C...-........CCTAAGCATCATATCAGA..  299
BG55821 (fast)     ........................................------------------..  282
HarMX2 construct   ........................................------------------..  282
MX2.3 PCR product  ......................A....C...-........CCTAAGCATCATATCAGA..  299

NT_033779          TTTTACTTACCCCGATTTGTGATCTCGTTGCCGGCATTACGATTGTTCTCGTCCTGGCCC  342
BDSC 55821         ............................................................  359
BG55821 (fast)     ............................................................  342
HarMX2 construct   ............................................................  342
MX2.3 PCR product  ............................................................  359

NT_033779          CCCATGCTGACCGTCTTTGACTTTCTGGGCACCGACGTCTCATCCTCCTCGTCCTCGTCG  402
BDSC 55821         ............................................................  419
BG55821 (fast)     ............................................................  402
HarMX2 construct   ............................................................  402
MX2.3 PCR product  ............................................................  419

NT_033779          ACATCCTCGCCCGTTTCCACGCCGTCGTCCTCCTCATCATCGGCGAGAGCTGATTGCGTG  462
BDSC 55821         ............................................................  479
BG55821 (fast)     ............................................................  462
HarMX2 construct   ............................................................  462
MX2.3 PCR product  ............................................................  479

NT_033779          GTGGTCAGAGGCGAACCAGCGGTCTTCGTGGAGCTGGGA                       501
BDSC 55821         .................mmmmmmmmmmmmmmmmmmmmmm                       496
BG55821 (fast)     ................mmmmmmmmmmmmmmmmmmmmmmm                       478
HarMX2 construct   .................................GTTACA                       501
MX2.3 PCR product  .......................................                       518
```

Figure S2B

```
NT_033779          ATTCGAAACCGCTACTCTGGCTCGGCCACAAAGTGGGCTTGGTCGCTGTTGCGGACAAGT   60
BDSC 55821         ............................................................   60
BG55821 (fast)     ............................................................   60
HarMX5 construct   TAGAAAGTATAGGAACTTC.........................................   60
MX5.1 PCR product  TAGAAAGTATAGGAACTTC.........................................   60

NT_033779          GAGATTGCTAATGAGCTGCTTTTAGGGGGCGTGTTGTGCTTGCTTTCCAACTTTTCTAGA  120
BDSC 55821         ............................................................  120
BG55821 (fast)     ............................................................  120
HarMX5 construct   ............................................................  120
MX5.1 PCR product  ............................................................  120

NT_033779          TTGATTCTACGCTGCCTCCAGCAGCCACCCCTCCCATCA------CCATTCCCATCACCA  174
BDSC 55821         .......................................------....C..........  174
BG55821 (fast)     ................................T.....CTCATCC....C..........  180
HarMX5 construct   ................................T.....CTCATCC....C..........  180
MX5.1 PCR product  .......................................------....C..........  174

NT_033779          TCCAGTCCCGTTGGCTCCCAGTCACAGTATTACACGTATGCAAATTAAGCCGAAGTTCAA  234
BDSC 55821         ............................................................  234
BG55821 (fast)     ............................................................  240
HarMX5 construct   ............................................................  240
MX5.1 PCR product  ............................................................  234

NT_033779          TTGCGACCGCAGCAACAACACGATCTTTCTACACTTCTCCTTGCTATGCTTGACATTCAC  294
BDSC 55821         ............................................................  294
BG55821 (fast)     ............................................................  300
HarMX5 construct   ............................................................  300
MX5.1 PCR product  ............................................................  294

NT_033779          AAGGTCAAAGCTCTTAATATTCTGGCTCGTGGCCCTACACTGTAAGAAATTACTATAGAA  354
BDSC 55821         ............................................................  354
BG55821 (fast)     ............................................................  360
HarMX5 construct   ............................................................  360
MX5.1 PCR product  ............................................................  354

NT_033779          ATAACGGTACACGGAATAAGATATTTTTTTTAGTCCATATGCTTTTAACAAATGTGTTTT  414
BDSC 55821         ............................................................  414
BG55821 (fast)     ........................................A...................  420
HarMX5 construct   ........................................A...................  420
MX5.1 PCR product  ............................................................  414

NT_033779          AAGTTTATGTTATATTATTGTTAGAAAACCGGTGTTTTTTTA-AAATCGGTTAAAAAATT  473
BDSC 55821         G............................A...........TT.................  474
BG55821 (fast)     ..........................................-.................  479
HarMX5 construct   ..........................................-.................  479
MX5.1 PCR product  G............................A...........TT.................  474

NT_033779          ACTACGAGAGAAAAATACAAATTTTGTAAATAAGATTGACTCTTTTTCGATTTTGGAATA  533
BDSC 55821         ............................................................  534
BG55821 (fast)     ............................................................  539
HarMX5 construct   .........................................................GTT  539
MX5.1 PCR product  ............................................................  534

NT_033779          TTT                                                           536
BDSC 55821         ...                                                           537
BG55821 (fast)     ...                                                           542
HarMX5 construct   ACA                                                           542
MX5.1 PCR product  ...                                                           537
```

Figure S2C

```
NT_033779          TCATAATTTTTTGGTTTCAGGACCCTCTTTACTATTCCCAAAACGGGGTAGCTGTGATGG   60
BDSC 55821         ..T...A......C...........C..................................   60
BG55821 (fast)     ............................................................   60
HarMX6 construct                                                       CCCTGAAT    8
MX6.1 PCR product  ..T...A......C...........C..................................   60

NT_033779          CGGTTTGTTGTGGTCCTACAAGGGTCTTTGTGGACCGGGAACTGAAAGCGTTTTTGGAAT  120
BDSC 55821         ............................................................  120
BG55821 (fast)     ............................................................  120
HarMX6 construct   TCGCATCTAGA.................................................   68
MX6.1 PCR product  ............................................................  120

NT_033779          ACGGACAATCCTTTGCCGATCGCCTGCGGCGAGCGCCCTGCCAATCGACATCGGTTTGTG  180
BDSC 55821         ............................................................  180
BG55821 (fast)     ............................................................  180
HarMX6 construct   ............................................................  128
MX6.1 PCR product  ............................................................  180

NT_033779          GTCAGAATATTGTCAATGCCATCGAGAGATCGGAGAATGGTCAGATATGGATTGCGGATA  240
BDSC 55821         ............................................................  240
BG55821 (fast)     ............................................................  240
HarMX6 construct   ............................................................  188
MX6.1 PCR product  ............................................................  240

NT_033779          AGGGTGGACTCGAGTTGGTCAAATTGCATTGGTACTGGCACATGGCCGACCAGTTCGTGC  300
BDSC 55821         ..........G.................................................  300
BG55821 (fast)     ..........C.................................................  300
HarMX6 construct   ..........C.................................................  248
MX6.1 PCR product  ..........C.................................................  300

NT_033779          ACTATATGCAGAGCAATGATGAAGAGGATCAAGATTGAATTCGAATCAAATAAAATAATG  360
BDSC 55821         ............................................................  360
BG55821 (fast)     ............................................................  360
HarMX6 construct   ............................................................  308
MX6.1 PCR product  ............................................................  360

NT_033779          CTTTACGCAAAAAGTAGGCAATTCATTTTCCTATGATAATAGATAT--------------  406
BDSC 55821         ..............................................GGGTCATCTATGGG  420
BG55821 (fast)     ..............................................GGGTCATCTATGGG  420
HarMX6 construct   ..............................................GGGTCATCTATGGG  368
MX6.1 PCR product  ..............................................GGGTCATCTATGGG  420

NT_033779          --------------------------------------------TTTTTTTTTTTTTTTT  422
BDSC 55821         GTGTGAAAGAGTAATGACAAAATTTGGTGTGCCCAAAAGTATGC----------------  464
BG55821 (fast)     GTGTGAAAGAGTAATGACAAAATTTGGTGTGCCCAAAAGTATGC----------------  464
HarMX6 construct   GTGTGAAAGAGTAATGACAAAATTTGGTGTGCCCAAAAGTATGC----------------  412
MX6.1 PCR product  GTGTGAAAGAGTAATGACAAAATTTGGTGTGCCCAAAAGTATGC----------------  464

NT_033779          TTTTTTTTTTTTTTTTTTTTTGATGATGTTTTTATTATTTGATCAACGCACTGTTACCCA  482
BDSC 55821         ------------------------------------------------------------  464
BG55821 (fast)     ------------------------------------------------------------  464
HarMX6 construct   ------------------------------------------------------------  412
MX6.1 PCR product  ------------------------------------------------------------  464

NT_033779          TGCGGCAACTTAATTTGATCTGCTTAAATTATTTTATTTTACTATGTGTCTTGGTTACTT  542
BDSC 55821         ------------------------------------------------------------  464
BG55821 (fast)     ------------------------------------------------------------  464
HarMX6 construct   ------------------------------------------------------------  412
MX6.1 PCR product  ------------------------------------------------------------  464

NT_033779          AAGACTAACAGATTTTTAATCCTAAAAATGATAGGGAGAATTATAATAGTTGATATAACA  602
BDSC 55821         ------------------------------------------------------------  464
```

```
BG55821 (fast)     ------------------------------------------------------------  464
HarMX6 construct   ------------------------------------------------------------  412
MX6.1 PCR product  ------------------------------------------------------------  464

NT_033779          TAGTATTCTAATGAATTTTAAAACTAAGACTTTCTAGGTCAAAACCAGGTTAGGGAGGTC  662
BDSC 55821         ------------------------------------------------------------  464
BG55821 (fast)     ------------------------------------------------------------  464
HarMX6 construct   ------------------------------------------------------------  412
MX6.1 PCR product  ------------------------------------------------------------  464

NT_033779          ATGAGGGTGGTGTCGCTTGAGTCTTCTTTTGACTCTAGTCCGAGGGTCAGCATTGACCAC  722
BDSC 55821         ------------------------------------------------------------  464
BG55821 (fast)     ------------------------------------------------------------  464
HarMX6 construct   ------------------------------------------------------------  412
MX6.1 PCR product  ------------------------------------------------------------  464

NT_033779          AGCTGCAGTTCCTAGCTTCCTTGCTAGGCTGTTGGGGTGTACATTAAGCCTTTCCATATA  782
BDSC 55821         ------------------------------------------------------------  464
BG55821 (fast)     ------------------------------------------------------------  464
HarMX6 construct   ------------------------------------------------------------  412
MX6.1 PCR product  ------------------------------------------------------------  464

NT_033779          TTTTTGGGCGAGCGAATGTTGATGGGAGCTATAATTAGATGTGCTTAATTATGATGGGGT  842
BDSC 55821         ----------..................................................  514
BG55821 (fast)     ----------..................................................  514
HarMX6 construct   ----------.........................................GAAGTTCCT  462
MX6.1 PCR product  ----------.........................................GAAGTTCCT  514
```

Figure S2D

```
NT_033779            TTGCAGCCCAACTAATTAACATATAAATTTAGAGGTGTTGCACTTTTGTATTTATTTATG   60
BDSC 55821           mmmmmmmmmmmmmmmmmmmmmmmmmmmmmm..............................   30
BG55821 (fast)       mmmmmmmmmmmmmmmmmmmmmmmmm...................................   60
HarMX10 construct    TCTAGA......................................................   60
MX10.1 PCR product   mmmm........................................................   60

NT_033779            AATGAAACTGACAGAGACAAAGAGACGGCCTGGTCGTGGAGGTGGAGAAGGAGGCTGGCA  120
BDSC 55821           ...........................G................................   90
BG55821 (fast)       ...........................A................................  120
HarMX10 construct    ...........................A................................  120
MX10.1 PCR product   ...........................A................................  120

NT_033779            GGGAGTGGGAAAGTTTCAAGTGCACTGAGAAAAATTTTAAGTAAATTAAAAGCAATATTT  180
BDSC 55821           ............................................................  150
BG55821 (fast)       ............................................................  180
HarMX10 construct    ............................................................  180
MX10.1 PCR product   ............................................................  180

NT_033779            TGCGTATACAAATTGCTCATACATATAATCCTCAAAAAATGAATTGTTTTCGTTAACTCA  240
BDSC 55821           ............................................................  210
BG55821 (fast)       ............................................................  240
HarMX10 construct    ............................................................  240
MX10.1 PCR product   ............................................................  240

NT_033779            AAAGAATGTTACAATATGTACTGCCTAACTTATGTTTGGCATTTTGCATTTTTTGTCAAC  300
BDSC 55821           ........................C...................T...............  270
BG55821 (fast)       ........................T...................-...............  299
HarMX10 construct    ........................T...................-...............  299
MX10.1 PCR product   ........................T...................-...............  299

NT_033779            TTTTTCTGTCAGTGTGCAAATCTGCAATATGCATCTCGTACTCATATGCAAGCCAAGGTC  360
BDSC 55821           ............................................................  330
BG55821 (fast)       ............................................................  359
HarMX10 construct    ............................................................  359
MX10.1 PCR product   ............................................................  359

NT_033779            GATTTCTGTTCCGTTCAACTGCAGCAGCAGCAGCAGCAGCATCGACAACAGCGGATTGCA  420
BDSC 55821           ..................................................G.........  390
BG55821 (fast)       ..................................................A.........  419
HarMX10 construct    ..................................................A.........  419
MX10.1 PCR product   ..................................................A.........  419

NT_033779            GTTTATGGGAAAGAGTTTAGCCAGCCAAGATTTATTGGCAT                      461
BDSC 55821           .........................................                      431
BG55821 (fast)       .........................................                      460
HarMX10 construct    ................................GAAGTTCCT                      460
MX10.1 PCR product   ................................GAAGTTCCT                      460
```

Figure S2. Sequence alignments of the homology arm region facing *Adh* for each MX insertion line. This part of the insertion is relevant because it contains sequence that could have recombined in from the homology construct and that would be retained after tandem duplication (yellow). The distal homology arms would not be retained and so were not considered. As described in Results > contamination of homology arm sequence, the homology arms derive from the Adh^{fast} haplotype of strain BG-55821, but each construct inserted into the Adh^{slow} haplotype that matches strain BDSC 55821. Sequences are presented in left to right order that matches Figure 2.

> Yellow designates sequence mismatch between BDSC 55821 (injected strain) and MX marker-FRT insertion line in the homology arm region.
> **Bold** designates each guide RNA site including protospacer adjacent motif.
> Cyan designates the proximal segment of the 48bp FRT site. This is the functional end of the insertion.
> Magenta designates the start of the plasmid assembly connector. This part of the construct is not expected to recombine into the genome.
> "." designates sequence that matches the reference.
> "-" is an alignment gap.
> "m" designates missing data due to low read quality at the start of the Sanger sequence read. We checked all such sites in the DSPR whole-genome assemblies and none contained sequence polymorphisms.
> 'N' refers to an ambiguous base call in one BG55821 sequence read. This read was low quality but clearly matched MX10 at all four variant bases.

A) Alignment of MX2 right homology arm region. Several fast haplotype sequence variants occur in the construct's homology arm, but did not recombine into insertion line MX2.3.
B) Alignment of MX5 right homology arm region. Several fast haplotype sequence variants occur in the construct's homology arm, but did not recombine into insertion line MX5.1.
C) Alignment of MX6 left homology arm region. The one *fast* haplotype sequence variant that occurs in the construct's homology arm did recombine into insertion line MX6.1 (yellow). This is a synonymous variant in the last coding exon of *Adhr.* It is part of an XhoI site (CTCGAG) which is absent in BDSC 55821 (CTGGAG). In the alignment, we included some sequence to the left of the homology arm to illustrate that sequence variants outside of the homology arm region do match BDSC 55821. The large gap is a transposable element fragment that is specific to the reference genome and so is not relevant.
D) Alignment of MX10 left homology arm region. Four *fast* haplotype sequence variants occur in the construct's homology arm, each of which incorporated into insertion line MX10.1 (yellow). These all occur in noncoding sequence.

Sanger sequences were generated as follows. Homology arm construct sequences (plasmids) were determined using multiple primers (File S1). PCR product from BDSC 55821 and BG55821 was generated as described in Methods > Sequence Verification of Insertions. PCR product from strain MX2.3 was using primers -4500-1F/-4500-1R then sequenced with primers uwseq-F6 and -4500-1R. PCR product from strain MX5.1 was amplified and then sequenced with primers uwseq-F6/Adhseq-0408R. PCR product from strain MX6.1 was amplified and then sequenced with primers melst1896F/uwseq-R6. PCR product from strain MX10.1 was amplified using primers 12500-1F/12500-1R and then sequenced with primers 12500-1F and uwseq-R6.
We note that each PCR uses at least one primer outside of the homology constructs. This ensures that the sequence data is obtained from the gene-proximal insertion site even in the case of multiple marker-FRT insertions.
